# Supplementary figures and images for: Quantitative input–output dynamics of a c-di-GMP signal transduction cascade in Vibrio cholerae
Source: PLoS Biol. 2022 Mar 18;20(3):e3001585. doi: 10.1371/journal.pbio.3001585 (PMC8967002; doi:10.1371/journal.pbio.3001585)

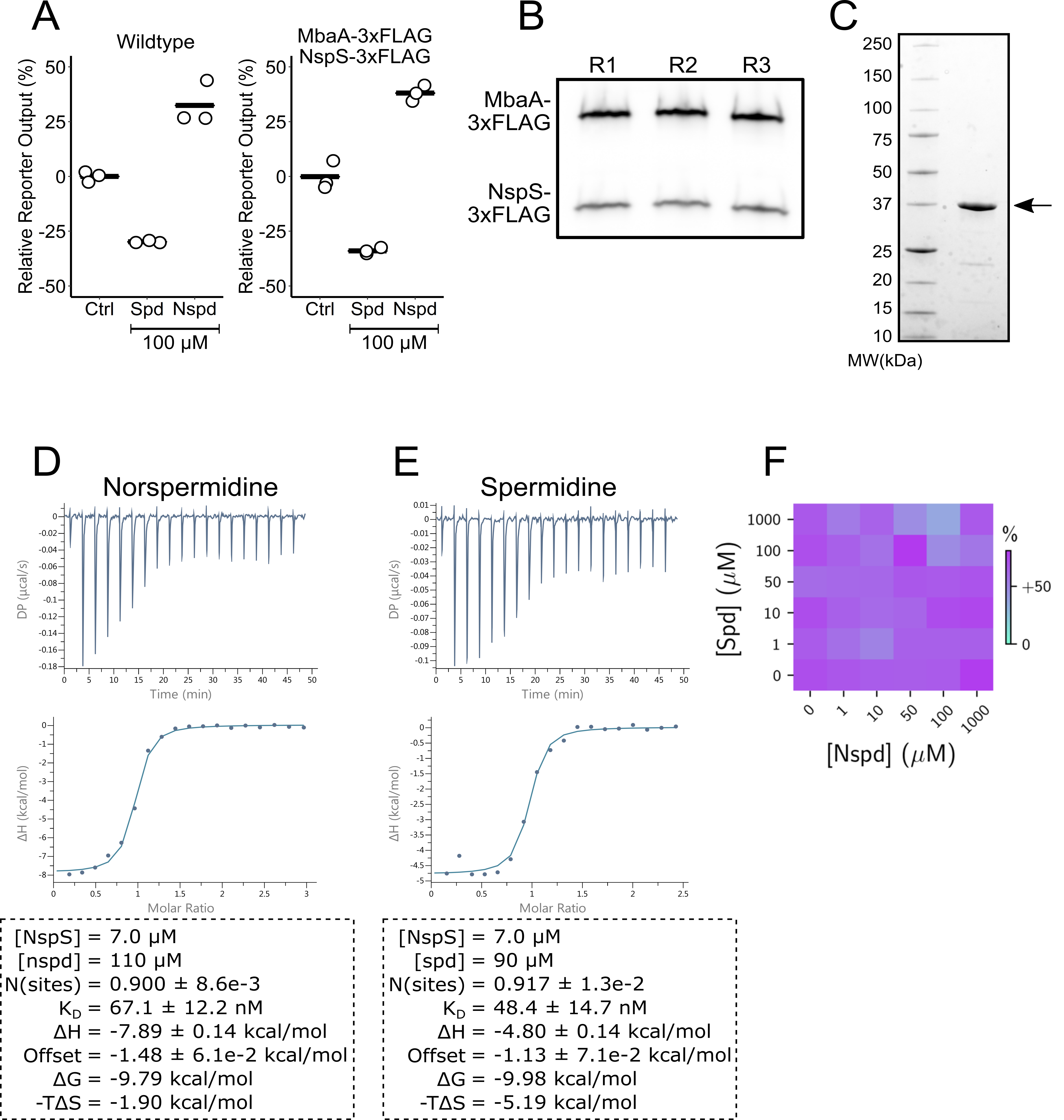

Supplement: S1 Fig — (A) Relative c-di-GMP reporter output for wild-type V. cholerae (left panel) and V. cholerae harboring mbaA-3xFLAG and nspS-3xFLAG (right panel) expressed from the native locus on the chromosome. Treatments: no addition (designated Ctrl), 100 μM spermidine, and 100 μM Nspd. (B) Western blot of MbaA-3xFLAG and NspS-3xFLAG. R1, R2, and R3 designate 3 biological replicates. (C) SDS-PAGE gel showing purity of the NspS-6xHis protein used for ITC measurements. Molecular weight markers are designated on the left. The arrow on the right shows the position of NspS-6xHis. (D) ITC data, plot, and calculated values for Nspd binding to purified NspS-6xHis. (E) As in D for Spd. (F) Shown is the c-di-GMP reporter output for a V. cholerae strain carrying Ptac-nspS-mbaA at the native nspS-mbaA locus. Data are displayed as percent increases relative to the wild-type strain with no polyamines added. Numerical values for plots are available in S1 Data. c-di-GMP, cyclic diguanylate; ITC, isothermal titration calorimetry; Nspd, norspermidine; Spd, spermidine. (TIF) [file pbio.3001585.s001.tif]

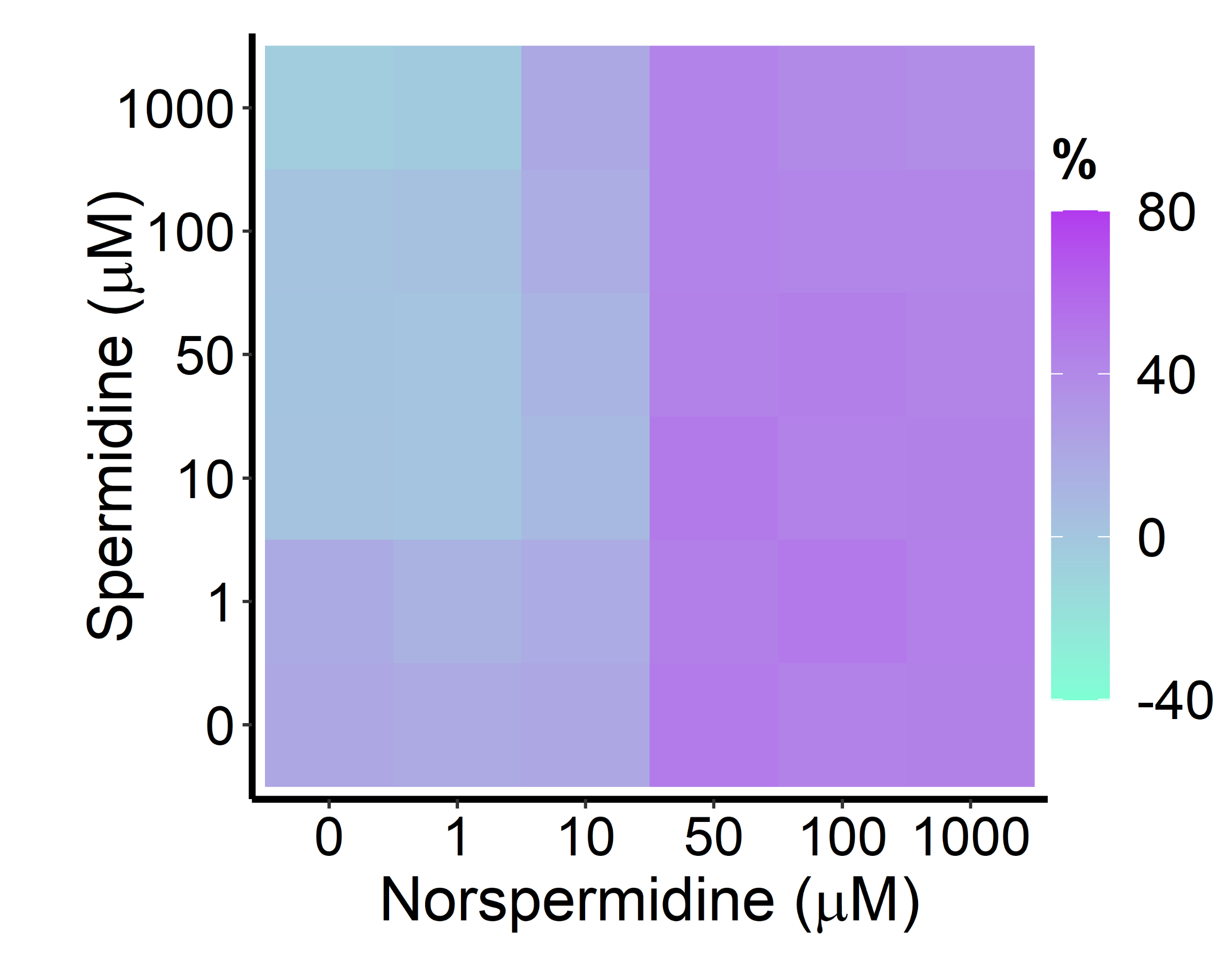

Supplement: S6 Fig — Experimentally obtained results for c-di-GMP reporter output in V. cholerae carrying Pbad-vpvCW240R treated with 0.0125% arabinose, for the indicated polyamine concentrations, displayed as a heatmap. Throughout the manuscript, data in c-di-GMP output heatmaps are displayed as percent differences compared to the untreated wild-type strain, with teal representing low and purple representing high c-di-GMP reporter output, respectively. Numerical values for plots are available in S1 Data. c-di-GMP, cyclic diguanylate. (TIF) [file pbio.3001585.s006.tif]

**Figure S1B**  
anti-FLAG

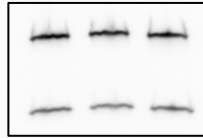

**Figure S1C**  
Purified NspS

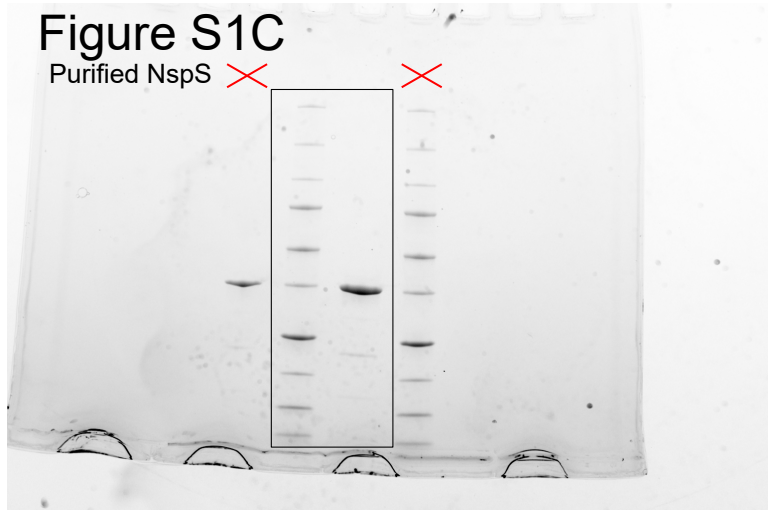

**Figure S2**  
anti-FLAG

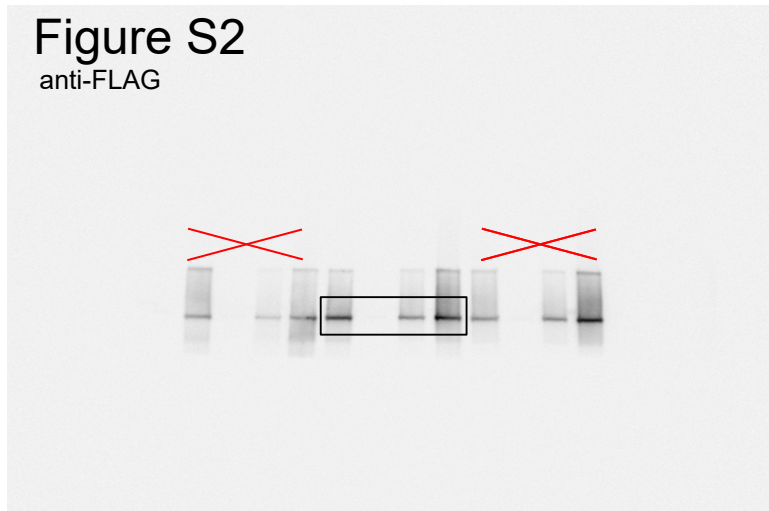

**Figure S2**  
anti-RpoA

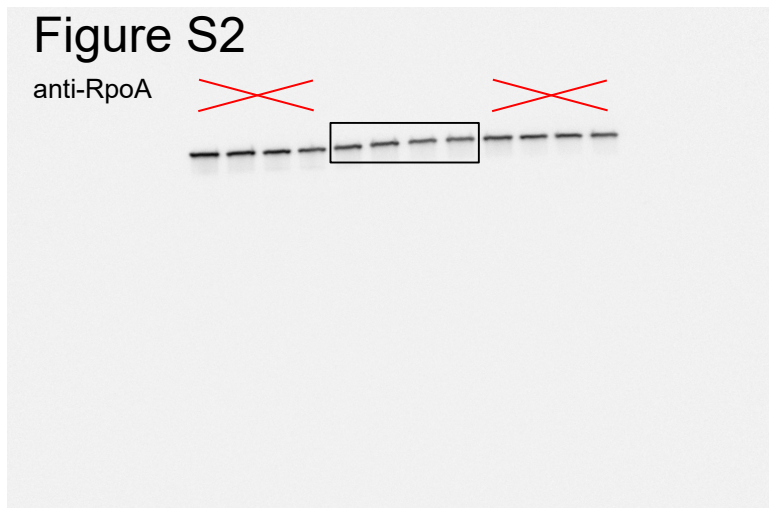

Supplement: S1 Raw Images — (PDF) [file pbio.3001585.s022.pdf]
