# Supplementary material for: Quantitative input–output dynamics of a c-di-GMP signal transduction cascade in Vibrio cholerae
Source: PLoS Biol. 2022 Mar 18;20(3):e3001585. doi: 10.1371/journal.pbio.3001585 (PMC8967002; doi:10.1371/journal.pbio.3001585)
Supplement: S1 Text — (PDF) [file pbio.3001585.s007.pdf]

Supplementary model for

Quantitative input-output dynamics of a c-di-GMP signal-transduction cascade in *Vibrio cholerae*

Andrew A. Bridges[1*], Jojo A. Prentice[1*], Chenyi Fei[1,2], Ned S. Wingreen[1,2#], Bonnie L. Bassler[1,3#]

[*]These authors contributed equally
[#]Correspondence to: wingreen@princeton.edu bbassler@princeton.edu

Affiliations:
[1]Department of Molecular Biology, Princeton University, Princeton, New Jersey 08544, USA.
[2]Lewis-Sigler Institute for Integrative Genomics, Princeton University, Princeton, New Jersey 08544, USA.
[3]The Howard Hughes Medical Institute, Chevy Chase, MD 20815, USA.

1 Notation

Throughout this document, we adopt the convention that species concentrations are denoted by the first letter of their names (i.e., [NspS] $\equiv N$). All energies are in units of the thermal energy $k_{\rm B}T$.

2 Free-energy model for MbaA activity

We assume that each MbaA homodimer is a two-state receptor, which can exist in either the diguanylate cyclase or phosphodiesterase state. According to equilibrium statistical mechanics, the average phosphodiesterase activity of MbaA, $\langle A_{\rm PDE}\rangle$, is equal to its probability of being in the conformation that corresponds to the phosphodiesterase state, given by

$$\langle A_{\rm PDE}\rangle = \frac{1}{1+\exp(f_{\rm MbaA})}, \tag{1}$$

where $f_{\rm MbaA}$ denotes the free-energy difference between the phosphodiesterase and diguanylate cyclase states. Likewise, the probability that MbaA is in the diguanylate cyclase state is $\langle A_{\rm DGC}\rangle = 1-\langle A_{\rm PDE}\rangle$. The three possible configurations for MbaA and their associated free energies are 1) phosphodiesterase and unbound to NspS, $E_{\rm PDE}$, 2) diguanylate cyclase and unbound to NspS, $E_{\rm DGC}$, and 3) diguanylate cyclase and bound to NspS, $E_{\rm DGC} - \log(N_{\rm free}^{\rm closed}/K_{\rm MbaA})$, where $K_{\rm MbaA}$ is the binding constant of diguanylate-cyclase-state MbaA for closed NspS. In the final expression, we assume that an NspS molecule exists in one of two conformations: closed, which can bind MbaA, or open, which cannot bind MbaA. Moreover, we consider only *free* NspS in the closed conformation, as the concentration of MbaA may be sufficient to titrate a significant fraction of NspS out of the free pool. The sum of the Boltzmann factors associated with configurations 2 and 3 is

$$\exp[-E_{\rm DGC}] + \exp\left[-E_{\rm DGC} + \log\frac{N_{\rm free}^{\rm closed}}{K_{\rm MbaA}}\right] = \exp\left[-E_{\rm DGC} + \log(1+\frac{N_{\rm free}^{\rm closed}}{K_{\rm MbaA}})\right].$$

Therefore, the apparent free energy of the MbaA diguanylate-cyclase state is $f_{\rm MbaA}^{\rm DGC} = E_{\rm DGC} - \log(1+\frac{N_{\rm free}^{\rm closed}}{K_{\rm MbaA}})$. The apparent free energy of the MbaA phosphodiesterase state is simply $f_{\rm MbaA}^{\rm PDE} = E_{\rm PDE}$. Using the equations for the free energies of each conformation, the free-energy offset, $f_{\rm MbaA}$, is given by

$$f_{\rm MbaA} = f_{\rm MbaA}^{\rm PDE} - f_{\rm MbaA}^{\rm DGC} = \epsilon_{\rm MbaA} + \log(1+\frac{N_{\rm free}^{\rm closed}}{K_{\rm MbaA}}), \tag{2}$$

where $\epsilon_{\rm MbaA} = E_{\rm PDE} - E_{\rm DGC}$.

Next, we find an explicit formulation for $N_{\rm free}^{\rm closed}$. We assume that norspermidine only binds the closed conformation of NspS ($K_{\rm nspd}^{\rm open} \gg K_{\rm nspd}^{\rm closed}$) and that spermidine only binds the open conformation of NspS ($K_{\rm spd}^{\rm closed} \gg K_{\rm spd}^{\rm open}$), where $K_{\rm nspd}^{\rm closed}$, $K_{\rm nspd}^{\rm open}$, $K_{\rm spd}^{\rm closed}$, and $K_{\rm nspd}^{\rm open}$ are the binding constants of norspermidine/spermidine for closed/open NspS. Therefore, we eliminate the superscripts in the binding constant nomenclature so that $K_{\rm spd}^{\rm open} = K_{\rm spd}$ and $K_{\rm nspd}^{\rm closed} = K_{\rm nspd}$. The four possible configurations for NspS and their associated free energies are 1) open with no ligand bound, $E_{\rm open}$, 2) closed with no ligand bound, $E_{\rm closed}$, 3) open with spermidine bound, $E_{\rm open} - \log(s_{\rm peri}/K_{\rm spd})$, and 4) closed with norspermidine bound, $E_{\rm closed} - \log(n_{\rm peri}/K_{\rm nspd})$, where $x_{\rm peri}$ denotes the periplasmic concentration of species x. The sums of Boltzmann factors for the closed and open states, respectively, are given by

$$\exp[-E_{\rm closed}] + \exp\left[-E_{\rm closed} + \log(n_{\rm peri}/K_{\rm nspd})\right] = \exp\left[-E_{\rm closed} + \log(1+n_{\rm peri}/K_{\rm nspd})\right],$$
$$\exp[-E_{\rm open}] + \exp\left[-E_{\rm open} + \log(s_{\rm peri}/K_{\rm spd})\right] = \exp\left[-E_{\rm open} + \log(1+s_{\rm peri}/K_{\rm spd})\right].$$

Therefore, the apparent free energy of the closed state is $f^{\text{closed}}_{\text{NspS}} = E_{\text{closed}} - \log(1+n_{\text{peri}}/K_{\text{nspd}})$, and the apparent free energy of the open state is $f^{\text{open}}_{\text{NspS}} = E_{\text{open}} - \log(1 + s_{\text{peri}}/K_{\text{spd}})$. Using these equations, the free-energy offset between the open and closed states, f_{NspS}, is

$$f_{\text{NspS}} = f^{\text{open}}_{\text{NspS}} - f^{\text{closed}}_{\text{NspS}} = \epsilon_{\text{NspS}} + \log\left(\frac{1 + \frac{n_{\text{peri}}}{K_{\text{nspd}}}}{1 + \frac{s_{\text{peri}}}{K_{\text{spd}}}}\right), \tag{3}$$

where $\epsilon_{\text{NspS}} = E_{\text{open}} - E_{\text{closed}}$. Based on this free-energy offset, the concentration of free NspS in the closed conformation is

$$N^{\text{closed}}_{\text{free}} = p_{\text{closed}} N_{\text{free}} = \frac{\exp(f_{\text{NspS}})}{1 + \exp(f_{\text{NspS}})} N_{\text{free}}. \tag{4}$$

Our data suggest that NspS and MbaA are stoichiometric ($N \approx M$, where M is the concentration of MbaA). Thus, if p_{bound} represents the probability of NspS being bound by MbaA, Z represents the canonical partition function for MbaA, and $R \equiv M/N$, N_{free} is given by

$$N_{\text{free}} = N(1 - p_{\text{bound}}) = N\left(1 - \frac{\exp(-E_{\text{DGC}} + \log \frac{N^{\text{closed}}_{\text{free}}}{K_{\text{MbaA}}})R}{Z}\right) = N\left(1 - \frac{R e^{\epsilon_{\text{MbaA}}} \frac{N^{\text{closed}}_{\text{free}}}{K_{\text{MbaA}}}}{e^{\epsilon_{\text{MbaA}}}(\frac{N^{\text{closed}}_{\text{free}}}{K_{\text{MbaA}}} + 1) + 1}\right). \tag{5}$$

Substituting the positive root of the solution to (4) and (5) into (2) gives the explicit formulation of the free-energy offset between the phosphodiesterase and diguanylate-cyclase states of MbaA.

3 Background on the Levenberg-Marquardt algorithm

The Levenberg-Marquardt algorithm belongs to a class of iterative algorithms that aim to solve the problem

$$\begin{aligned} \text{minimize} \quad & S(\boldsymbol{x}) = \sum_{i=1}^{m} r_i(\boldsymbol{x})^2 \\ \text{subject to} \quad & \boldsymbol{x}_{\text{min}} \leq \boldsymbol{x} \leq \boldsymbol{x}_{\text{max}}, \end{aligned} \tag{6}$$

where $\boldsymbol{x} \in \mathbb{R}^n$ is the vector of parameter values, $r_i : \mathbb{R}^n \to \mathbb{R}$ are the nonlinear residuals for each of the m data points, and $\boldsymbol{x}_{\text{min}}$ and $\boldsymbol{x}_{\text{max}}$ are the bounds on the parameter values (Levenberg 1944; Marquardt 1963; Press et al. 1988). Each $k+1$ iteration aims to find some perturbation, $\boldsymbol{\delta} = \boldsymbol{x}_{k+1} - \boldsymbol{x}_k$, to reduce S from the previous iteration. Newton's method minimizes the second-order Taylor expansion of $S(\boldsymbol{x})$. Beginning with an initial estimate of the parameter values, $\boldsymbol{x}_0$, the iteration proceeds as

$$\boldsymbol{x}_{k+1} = \boldsymbol{x}_k - (J(\boldsymbol{x}_k)^T J(\boldsymbol{x}_k) - U(\boldsymbol{x}_k))^{-1} J(\boldsymbol{x}_k)^T r(\boldsymbol{x}_k), \tag{7}$$

where $J(\boldsymbol{x}_k)$ denotes the Jacobian of the residuals with respect to the parameters at the kth iteration and

$$U(\boldsymbol{x}_k) = \sum_{i=1}^{m} r_i(\boldsymbol{x}_k) \nabla^2 r_i(\boldsymbol{x}_k)$$

is the second term in the Hessian $\nabla^2 S(\boldsymbol{x}_k) = J(\nabla(S(\boldsymbol{x}_k)))$, where ∇ denotes the vector differential operator. When the number of parameters, n, is small, Newton's method is suitable. However, the number of derivatives that need to be calculated in $U(\boldsymbol{x}_k)$ at each iteration is a quadratic function of n, which can become computationally expensive as the number of parameters increases. To circumvent this issue, if the residuals are locally small around the solution (approximately quadratic in the parameters), $U(\boldsymbol{x})$ may be neglected. This assumption is the basis for the Gauss-Newton algorithm, which iterates the parameters by

$$\boldsymbol{x}_{k+1} = \boldsymbol{x}_k - (J(\boldsymbol{x}_k)^T J(\boldsymbol{x}_k))^{-1} J(\boldsymbol{x}_k)^T r(\boldsymbol{x}_k), \tag{8}$$

or equivalently,

$$(J(\boldsymbol{x}_k)^T J(\boldsymbol{x}_k))\boldsymbol{\delta} = -J(\boldsymbol{x}_k)^T r(\boldsymbol{x}_k). \tag{9}$$

In certain scenarios, the size of the correction term $\boldsymbol{\delta}$ may be too large or the correction may veer in the wrong direction. In these cases, it helps to place a bound on $||\boldsymbol{\delta}||_2$; such methods are called trust region techniques (Moré and Sorensen 1983). The Levenberg-Marquardt algorithm achieves such a constraint through the use of an adaptive Lagrange multiplier, λ_k, giving the update rule

$$(J(\boldsymbol{x}_k)^T J(\boldsymbol{x}_k) + \lambda_k I)\boldsymbol{\delta} = -J(\boldsymbol{x}_k)^T r(\boldsymbol{x}_k), \tag{10}$$

where I denotes the identity matrix. For small λ_k, this algorithm is equivalent to the Gauss-Newton algorithm, but for large λ_k, $\boldsymbol{\delta}$ is small and moves in the direction of steepest descent (the method of gradient descent). λ is initialized at a large value so that the algorithm is equivalent to gradient descent in the first few steps. If an iteration results in an increase in $S(\boldsymbol{x})$, λ is increased. If the iteration decreases $S(\boldsymbol{x})$, λ is decreased so that the Levenberg-Marquardt algorithm approaches the Gauss-Newton algorithm. For further details on the convergence properties and time complexities of these algorithms, readers are referred to (Levenberg 1944; Marquardt 1963; Press et al. 1988).

References

Levenberg, Kenneth (1944). "A method for the solution of certain non-linear problems in least squares". *Quarterly of applied mathematics* 2.2, pp. 164–168.

Marquardt, Donald W (1963). "An algorithm for least-squares estimation of nonlinear parameters". *Journal of the society for Industrial and Applied Mathematics* 11.2, pp. 431–441.

Press, William H et al. (1988). *Numerical recipes in C.*

Moré, Jorge J and Danny C Sorensen (1983). "Computing a trust region step". *SIAM Journal on scientific and statistical computing* 4.3, pp. 553–572.
