# Supplementary material for: Quantitative input–output dynamics of a c-di-GMP signal transduction cascade in Vibrio cholerae
Source: PLoS Biol. 2022 Mar 18;20(3):e3001585. doi: 10.1371/journal.pbio.3001585 (PMC8967002; doi:10.1371/journal.pbio.3001585)
Supplement: S1 Table — (PDF) [file pbio.3001585.s008.pdf]

Table S1: Model Parameters

Symbol	Description	Value
N	Total concentration of NspS.	16.7 μM
R	Molar ratio of MbaA to NspS.	1
n_{ext}	Extracellular concentration of norspermidine.	Input
s_{ext}	Extracellular concentration of spermidine.	Input
K_{spd}	Dissociation constant of spermidine from NspS in the open conformation.	48.0 nM
K_{nspd}	Dissociation constant of norspermidine from NspS in the closed conformation.	0.5 nM
ϵ_{NspS}	Free-energy offset between the open and closed conformations of apo-NspS.	-4.87 $k_{\mathrm{B}}T$
ϵ_{MbaA}	Free-energy offset between the phosphodiesterase and diguanylate cyclase states of MbaA in the absence of NspS.	-1.214 $k_{\mathrm{B}}T$
K_{MbaA}	Dissociation constant of closed-conformation NspS from diguanylate-cyclase-state MbaA.	50 nM
ψ	Rate of norspermidine transport from the periplasm to the cytoplasm by PotD1.	*10.6/t
φ	Rate of spermidine transport from the periplasm to the cytoplasm by PotD1.	*5.7/t
P	Periplasmic concentration of PotD1.	10 μM
α	Rate of norspermidine production and export to the periplasm.	22.5 μM/t
β_n	Rate of diffusion of extracellular norspermidine across the outer membrane.	*5/t
β_s	Rate of diffusion of extracellular spermidine across the outer membrane.	*5/t
K^n_{PotD1}	Michaelis constant for PotD1-norspermidine.	1 nM
K^s_{PotD1}	Michaelis constant for PotD1-spermidine.	434 nM
γ	Background rate of c-di-GMP production.	20 μM/t
ν	Background rate of c-di-GMP degradation.	*1/t
μ	Rate of c-di-GMP degradation by MbaA.	*0.78/t
λ	Rate of c-di-GMP synthesis by MbaA.	7.8 μM/t

*In this table, parameter values with units of inverse time $1/t$ are denoted value$/t$, where t is an arbitrary unit of time.
