# Supplementary material for: Quantitative input–output dynamics of a c-di-GMP signal transduction cascade in Vibrio cholerae
Source: PLoS Biol. 2022 Mar 18;20(3):e3001585. doi: 10.1371/journal.pbio.3001585 (PMC8967002; doi:10.1371/journal.pbio.3001585)
Supplement: S2 Table — (DOCX) [file pbio.3001585.s009.docx]

**S2 Table. Strains used in this study.**

| Strain Number | Genotype | Plasmid | Antibiotic Resistance | Origin |
| --- | --- | --- | --- | --- |
| BB_Vc_0090 | Wildtype O1 El Tor biotype C6706str2 | - | Sm | Laboratory wildtype |
| AB_Vc_761 | Δ*vc1807*::Cm^R^ (Referred to as wildtype in this work) | - | Sm, Cm | Bridges et al. 2020 |
| AB_Vc_956 | Wildtype | pFY4357::Gm^R^ | Sm, Gm | Bridges et al. 2021 |
| AB_Vc_977 | Δ*nspS* Δ*vc1807*::Kan^R^ | pFY4357::Gm^R^ | Sm, Gm, Kan | Bridges et al. 2021 |
| AB_Vc_975 | Δ*mbaA* Δ*vc1807*::Kan^R^ | pFY4357::Gm^R^ | Sm, Gm, Kan | Bridges et al. 2021 |
| AB_Vc_969 | Δ*potD1* Δ*vc1807*::Cm^R^ | pFY4357::Gm^R^ | Sm, Gm, Cm | Bridges et al. 2021 |
| AB_Vc_1122 | *mbaA-3xFLAG nspS-3xFLAG*  Δ*vc1807*::Spec^R^ | - | Sm, Spec | NT of AB_Vc_835 |
| AB_Vc_1133 | *mbaA-3xFLAG nspS-3xFLAG*  Δ*vc1807*::Spec^R^ | pFY4357::Gm^R^ | Sm, Gm, Spec | Conj of AB_Vc_1122 |
| AB_Ec_084 | *E. coli* BL21 (DE3) | pET15B::*npsS-6xHis*::Amp^R^ | Amp | Transformation of BL21 |
| AB_Vc_835 | *mbaA-3xFLAG* Δ*vc1807*::Kan^R^ | - | Sm, Kan | Bridges et al. 2021 |
| AB_Vc_1182 | Δ*mbaA* Δ*vc1807*::*Pbad-mbaA-3xFLAG*::Spec^R^ Δ*lacIZ*::Kan^R^ | - | Sm, Spec, Kan | NT of AB_Vc_1181 |
| JP_Vc_1192 | Δ*mbaA* Δ*vc1807*::*Pbad-mbaA-3xFLAG*::Spec^R^ Δ*lacIZ*::Kan^R^ | pFY4357::Gm^R^ | Sm, Spec, Kan | Conj of AB_Vc_1182 |
| AB_Vc_960 | Δ*nspC* Δ*vc1807*::Kan^R^ | pFY4357::Gm^R^ | Sm, Gm, Kan | Conj of AB_Vc_823 |
| AB_Vc_994 | Δ*nspC* Δ*potD1* Δ*vc1807*::Kan^R^ | pFY4357::Gm^R^ | Sm, Gm, Kan | Conj of AB_Vc_993 |
| AB_Vc_993 | Δ*nspC* Δ*potD1* Δ*vc1807*::Kan^R^ | - | Sm, Kan | NT of AB_Vc_711 |
| AB_Vc_801 | Δ*vc1807*::Kan^R^ | pEVS143-*P_vpsL_-lux*::Cm^R^ | Sm, Cm, Kan | Bridges et al. 2020 |
| AB_Vc_1088 | *vpvc^W240R^* Δ*vc1807*::Spec^R^ | - | Sm, Spec | NT of BB_Vc_0090 |
| AB_Vc_839 | Δ*mbaA* Δ*vc1807*::Kan^R^ | - | Sm, Kan | Bridges et al. 2021 |
| AB_Vc_958 | Δ*vpsL* Δ*vc1807*::Kan^R^ | pFY4357::Gm^R^ | Sm, Kan | Conj of AB_Vc_487 |
| AB_Vc_962 | Δ*vpsL vpvC^W240R^* | pFY4357::Gm^R^ | Sm, Gm | Conj of BB_Vc_0101 |
| AB_Vc_1145 | *Ptac-nspS-mbaA-3xFLAG* Δ*vc1807*::Spec^R^ | - | Sm, Spec | NT of AB_Vc_835 |
| AB_Vc_1203 | Δ*vpsL Ptac-nspS-mbaA-3xFLAG* Δ*vc1807*::Kan^R^ | pFY4357::Gm^R^ | Sm, Gm, Kan | Conj of AB_Vc_1197 |
| AB_Vc_996 | Δ*cdgL* Δ*vc1807*::Kan^R^ | - | Sm, Kan | NT of BB_Vc_0090 |
| JP_Vc_1206 | Δ*cdgL* Δ*vc1807*::Kan^R^ | pFY4357::Gm^R^ | Sm, Gm, Kan | Conj of AB_Vc_996 |
| AB_Vc_1056 | Δ*vc1807*::*Pbad-cdgL*::Kan^R^ | - | Sm, Kan | NT of BB_Vc_0090 |
| AB_Vc_1104 | Δ*vc1807*::*Pbad-cdgL*::Kan^R^ | pFY4357::Gm^R^ | Sm, Gm, Kan | Conj of 1056 |
| AB_Vc_1124 | Δ*vpvC* Δ*vc1807*::Spec^R^ | - | Sm, Spec | NT of BB_Vc_0090 |
| JP_Vc_1205 | Δ*vpvC* Δ*vc1807*::Spec^R^ | pFY4357::Gm^R^ | Sm, Gm, Spec | Conj of AB_Vc_1124 |
| AB_Vc_1109 | Δ*vc1807*::*Pbad-vpvC^W240R^*::Kan^R^ | - | Sm, Kan | NT of BB_Vc_0090 |
| AB_Vc_1110 | Δ*vc1807*::*Pbad-vpvC^W240R^*::Kan^R^ | pFY4357::Gm^R^ | Sm, Gm, Kan | Conj of 1109 |
| JP_Vc_1296 | Δ*vpsL vpvC^W240R^* Δ*vc1807*::*Pbad-vpvC^W240R^*::Kan^R^ | pFY4357::Gm^R^ | Sm, Gm, Kan | Conj of 1110 |
| AB_Vc_971 | *mbaA(D426A,E427A)*-*3xFLAG* Δ*vc1807*::Kan^R^ | pFY4357::Gm^R^ | Sm, Gm, Kan | Conj of 870 |

NT = Natural Transformation

Conj = Conjugation
