# Supplementary material for: Quantitative input–output dynamics of a c-di-GMP signal transduction cascade in Vibrio cholerae
Source: PLoS Biol. 2022 Mar 18;20(3):e3001585. doi: 10.1371/journal.pbio.3001585 (PMC8967002; doi:10.1371/journal.pbio.3001585)
Supplement: S3 Table — (DOCX) [file pbio.3001585.s010.docx]

**S3 Table. DNA oligonucleotides and gene fragments used in this study.**

| Oligo # | Name | Purpose | Direction | 5' to 3' Sequence |
| --- | --- | --- | --- | --- |
| 691 | *npsS*_3000up | Cloning at *nspS* locus | F | GACTTTATCAGGCCTACTCGCGTTATCCCTG |
| 890 | *npsS_3xFlag*_B | Cloning *nspS-3xFLAG* | R | CGCTGCTCTGGGAGATAAATCGAGAGATC |
| 981 | *nspS*_*3xFLAG*_  GSLinker_Gblock | Cloning *nspS-3xFLAG* | F | GATCTCTCGATTTATCTCCCAGAGCAGCGATTGGCAGAAGGTATCATTGATTCTGAACTTTCTGCTAAGAATCTGTCTCTTCGTGCTAAGATTATCTCTTCAGTTACGTATCAGTACGAAGCGAAACCTTACGGTTCAGGAAGTGGTAGTGGATCTGACTACAAAGACCATGACGGTGATTATAAAGATCATGATATCGATTACAAGGATGACGATGACAAGTGATCAGCAGTGTGACTTACCAATATGAAGCTAAACCATAG |
| 891 | *npsS_3xFlag*_C | Cloning *nspS-3xFLAG* | F | CAGCAGTGTGACTTACCAATATGAAGCTAAACCATAG |
| 698 | *npsS_*3000dwn | Cloning at *nspS* locus | R | CGCGTGGTTCGAATGAGCTCAAGTC |
| 692 | *npsS_*2700up | Cloning at *nspS* locus | F | CTCTACAAAGCGGAACGTGGTTAACCG |
| 697 | *npsS_*2700dwn | Cloning at *nspS* locus | R | CCTAATGACATCACTGGCTAAGCCGAG |
| 862 | pET15B_RBS | *pET15b-nspS-6xHis* | R | catggtatatctccttcttaaagttaaacaaaattatttctagaggggaattg |
| 863 | pET15B_cleav6His | *pET15b-nspS-6xHis* | F | ggaagcagtggtagcggcctggtgcc |
| 864 | pET15B_*npsS* | *pET15b-nspS-6xHis* | F | gaaataattttgtttaactttaagaaggagatataccatggaactgaatgtctacctttgggaagatac |
| 865 | pET15B_*npsS* | *pET15b-nspS-6xHis* | R | ggcaccaggccgctaccactgcttcctggtttagcttcatattggtaagtcacactg |
| 577 | *mbaA_*100up | Cloning at *mbaA* locus | F | GAAACCTGACATTGCCGCAATCAATGC |
| 893 | *3xFLAG_*AbR | Δ*vc1807::Pbad-mbaA-3xFLAG::*Spec^R^ | R | GTCGACGGATCCCCGGAATTCACTTGTCATCGTCATCCTTGTAATCG |
| 986 | *vc1807_3xFLAG*_E | Δ*vc1807::Pbad-mbaA-3xFLAG::*Spec^R^ | F | CGATTACAAGGATGACGATGACAAGTGAATTCCGGGGATCCGTCGACCTGCAG |
| 230 | *vc1807_*100dwn | Δ*vc1807::Pbad-mbaA-3xFLAG::*Spec^R^ | R | GGTAAAGTCGTAGGCTCTGTCGCTG |
| 1011 | *nspS_*100dwn | Δ*vc1807::Pbad-mbaA-3xFLAG::*Spec^R^ | F | CACGCTGGCCAAAAGTGACATCATC |
| 233 | ABD124 | Δ*vc1807::Pbad-mbaA-3xFLAG::*Spec^R^ | R | TGTAGGCTGGAGCTGCTTC |
| 571 | *mbaA_*3000up | Endogenous *Ptac-nspS-mbaA* | F | GCGCGCTAATCTGAACTCAACCCATAAG |
| 1015 | *Ptac-nspS_*ENDO_B | Endogenous *Ptac-nspS-mbaA* | R | ctgacgccagaagcattggtgcaGATCCGACAAATAACCTAATAGCGTAAAAG |
| 1016 | *Ptac-nspS_*ENDO_C | Endogenous *Ptac-nspS-mbaA* | F | CTTTTACGCTATTAGGTTATTTGTCGGATCtgcaccaatgcttctggcgtcag |
| 971 | *nspS_3xFlag*_rev | Endogenous *Ptac-nspS-mbaA* | R | CTATGGTTTAGCTTCATATTGGTAAGTCACACTGCTG |
| 572 | *mbaA_*2700up | Endogenous *Ptac-nspS-mbaA* | F | CGTTAGCATTCCACGCGGTCAGTTAG |
| 890 | *npsS_3xFlag_*B | Endogenous *Ptac-nspS-mbaA* | R | CGCTGCTCTGGGAGATAAATCGAGAGATC |
| 878 | *cdgL_*3000up | Cloning at *cdgL* locus | F | CGCCGATTACCTTCAATCTCAAGGTATC |
| 881 | *cdgL_*B | Deleting *cdgL* | R | CAAATTTCAAATGATGTATCGTTTAAAATATCACCATCGTCATAATAAACCTTTAC |
| 882 | *cdgL_*C | Deleting *cdgL* | F | GTAAAGGTTTATTATGACGATGGTGATATTTTAAACGATACATCATTTGAAATTTG |
| 885 | *cdgL_*3000down | Cloning at *cdgL* locus | R | GTGAGTTTCGCTCGAACCTGCATCG |
| 879 | *cdgL_*2700up | Cloning at *cdgL* locus | F | GTTCGTTACTCGCATATCATCGATCCAACTAC |
| 884 | *cdgL_*2700down | Cloning at *cdgL* locus | R | GTAGCGGGTGTAGATCTGCTGCGTTC |
| 105 | BBC1881 | Δ*vc1807*::*Pbad-*cloning | F | TTTAAAGGGGATCAGTGACCG |
| 721 | *Pbad_vc1807*_  Universal_B | Δ*vc1807*::*Pbad-*cloning | R | catttcacacctcctgcaggtac |
| 911 | *Pbad_cdgL*_C | Δ*vc1807*::*Pbad-cdgL*::Kan^R^ | F | gtacctgcaggaggtgtgaaatgAATTTAAATAACTTTAGCCTACGCTGGCTAAC |
| 912 | *Pbad_cdgL*_D | Δ*vc1807*::*Pbad-cdgL*::Kan^R^ | R | GTCGACGGATCCCCGGAATTTAGACAAAATTTCGCACAACGTATCGATC |
| 232 | ABD123 | Δ*vc1807::Pbad-*cloning | F | ATTCCGGGGATCCGTCGAC |
| 106 | BBC1882 | Δ*vc1807*::*Pbad-*cloning | R | CAATTTTGCTTTTGGACCATCCC |
| 978 | *Pbad_vpvC_*fwd | Δ*vc1807*::*Pbad-vpvC^W240R^*::Kan^R^ | F | gtacctgcaggaggtgtgaaatgactgatcaaacgcgaacttcgc |
| 979 | *Pbad_vpvC_*rev | Δ*vc1807*::*Pbad-vpvC^W240R^*::Kan^R^ | R | GTCGACGGATCCCCGGAATctatctgaactgatcctgcttgagttctttcgc |
| 203 | *vpvC_*3000up | Cloning at *vpvC* locus | F | CGATCCAGTGCATGCAGCGGAAATTGC |
| 998 | *vpvC_*B | Deleting *vpvC* | R | GATGGCGAAAGTGCTCGAAGCCTCTCGCGAAGAGCACCAGTAATACTTTCAGCGCG |
| 999 | *vpvC_*C | Deleting *vpvC* | F | CGCGCTGAAAGTATTACTGGTGCTCTTCGCGAGAGGCTTCGAGCACTTTCGCCATC |
| 204 | *vpvC_*3000down | Cloning at *vpvC* locus | R | CCGGCTGATGCTTTGTGTCTAACGTGCTG |
| 997 | *vpvC_*2700up | Cloning at *vpvC* locus | F | CTCACTGCGGCTGGCATTAAAGTTTCTCTG |
| 1001 | *vpvC_*2700down | Cloning at *vpvC* locus | R | GCTCACTTTGGCACCTACCCTGATTATTG |
