# Supplementary material for: Quantitative input–output dynamics of a c-di-GMP signal transduction cascade in Vibrio cholerae
Source: PLoS Biol. 2022 Mar 18;20(3):e3001585. doi: 10.1371/journal.pbio.3001585 (PMC8967002; doi:10.1371/journal.pbio.3001585)
Supplement: Attachment — Submitted filename: PolyamineManuscript2022_ResponsetoReviewers.pdf [file pbio.3001585.s023.pdf]

Thank you for submitting your manuscript "Quantitative input-output dynamics for a c-di-GMP signal-transduction cascade in Vibrio cholerae" for consideration as a Research Article at PLOS Biology. Your manuscript has been evaluated by the PLOS Biology editors, an Academic Editor with relevant expertise, and by several independent reviewers.

In light of the reviews (below), we will not be able to accept the current version of the manuscript, but we would welcome re-submission of a much-revised version that takes into account the reviewers' comments. We cannot make any decision about publication until we have seen the revised manuscript and your response to the reviewers' comments. Your revised manuscript is also likely to be sent for further evaluation by the reviewers.

In particular, we think it is important that you improve the introduction and discussion to enhance the contextualization of the work, as Reviewer #1 suggests. Reviewer #2 questions why the chromosomal vpvC-W240R gene affect transcription of motility genes but expressing it in trans does not. This reviewer also thinks that you could examine the expression levels of the vpvC-W240R gene in the different backgrounds, show the colony biofilm phenotypes of the expression mutants and whether differential vps-W240R expression explain the different phenotypes, and quantify the absolute levels of c-di-GMP in key strains. Reviewer #3 wants you to test whether in a npsC/potD mutant the variation in level of c-di-GMP is observed, to test whether the response is c-di-GMP-dependent in the wild type, whether NspS bound to spermidine also binds at low frequency to MbaA and how will this be accounted for into the mathematical model, clarify the rationale behind your indication that MbaA unbound to NspS can also display diguanylate cyclase activity, and comment on how the NspS-MbaA system can be adapted to other c-di-GMP signaling systems. Reviewer #4 thinks that the key question would be what the local/pathway-specific effectors that could contribute to physiological polyamine sensing and related MbaA activities are. Please address all the reviewers' issues.

Dear Editor,

Thank you for considering our manuscript. We are pleased that the reviewers and editors found the topics we covered of interest. The reviewers kindly provided us suggestions to improve our manuscript. We have taken these suggestions to heart and revised the manuscript exactly as requested. Below are our point-by-point responses to the reviewers' comments.

Reviewer comments are in black text and our responses are in green text. References to line numbers in the revised manuscript correspond to the manuscript file in which track changes have been accepted (PolyamineManuscript2022_Revisions_Clean.doc). We also provide a document in which changes are highlighted (PolyamineManuscript2022_Revisions_Changes Highlighted.doc). We hope that the revised manuscript is now acceptable for publication in *PLOS Biology*. Please let us know if you need anything more.

Yours,

Bonnie Bassler

Reviewer #1:

The manuscript "Quantitative input-output dynamics for a c-di-GMP signal transduction cascade in Vibrio cholerae" describes further characterization of the NspS-MbaA pathway, which regulates biofilm formation and dispersal in response to specific polyamines. This pathway is of particular importance to the field of c-di-GMP signaling and biofilm formation as signals for this pathway have been identified making it possible to study all the steps of the signal transduction cascade from signal input to phenotypic output. The work sheds light on the long-standing issue of how signal specificity may be achieved in signal transduction pathways that use c-di-GMP as a second messenger.

This a beautiful and thorough study that investigates the NspS/MbaA pathway using a powerful and tunable genetic system. It's well written, clear, and concise. The authors build a mathematical model of signal processing by this pathway that accurately explains experimental observations of c-di-GMP levels under different nspd and spd concentrations and changes to MbaA levels. They show that the pathway is very sensitive to nspd with a dissociation constant in the subnanomolar range and 100-fold lower than that for spd. They demonstrate that high affinity import by PotABCD1 leads to very low periplasmic concentrations of nspd and spd, which is consistent with the sensitivity of the NspS/MbaA pathway to these polyamines. They show that this pathway regulates transcription of biofilm-specific genes and not others involved in other c-di-GMP regulated phenotypes such as motility. In addition, using both the NspS/MbaA and other c-di-GMP metabolizing proteins (VpvC(W240R) and CdgL), the authors show that c-di-GMP signaling specificity can be explained by changes in the cytoplasmic c-di-GMP levels and that local signaling is not required for specificity. However, they also demonstrate, local signaling most likely occurs with the NspS/MbaA system since vpsL gene expression is activated at nspd concentrations that do not elevate c-di-GMP pools.

We thank the reviewer for the positive comments and thoughtful suggestions. We address each point below:

Major issues

The authors should do a better job of putting current study in the context of previous work by other groups as well. Some of the observations/experiments reported here have been reported by other groups previously. In most of these cases, the techniques used in these earlier studies are different than and not as sensitive as those used in this study and the effects appear to be observed at higher concentrations. However, many of the observations are consistent with those reported in this study and strengthen the claims of the current study.

For example, some of the experiments described in fig 3 done with nspC and nspCpotD1 mutants have been reported before (Wotanis et al. 2017) and should be cited. While effect of spd was not measured and only biofilm assays were reported in this study, the response of the nspCpotD1 double mutant to nspd was clearly demonstrated in this publication and is consistent with results in the current study. The idea that "MbaA transmits information internally to elicit gene expression changes at polyamine concentrations below that required to change cytoplasmic c-di-GMP" levels" has also been described before (Sobe et al. 2017) and should be cited. Additionally, effect of nspd on vpsL transcription (Lines 317-318) has been reported (Karatan et. al, 2005). MbaA transcriptomic analysis has also been reported in the same publication and is consistent with the observation that a small set of biofilm-specific genes being affected by the NspS/MbaA pathway (lines 369-372) and should be cited. In cases where results of previous work are not consistent (lack of changes in c-di-GMP pools in response to nspd or in nspS and mbaA mutants, Sobe et al. 2017), an attempt should be made to provide possible explanations for the differences.

We thank the reviewer for catching these oversights. In response, we have added text discussing the connection between the current work and previous studies. For example, we now include the following statements and citations:

(line 267) “To eliminate both polyamine production and import, we constructed the Δ*nspC* Δ*potD1* double mutant, a strain that has previously been used to assess the effect of exogenously-supplied norspermidine on biofilm formation (22).”

(line 344) “Thus, the dichotomy is that *vps* gene expression and the resulting changes in the *V. cholerae* biofilm lifestyle are triggered at extracellular norspermidine and spermidine levels that have no measurable effects on global cytoplasmic c-di-GMP levels, consistent with previous results (18).”

(line 328) “NspS-MbaA detection of norspermidine and spermidine leads to induction and repression, respectively, of *vps* biofilm matrix genes (16,17).”

(line 385) “Collectively, these data demonstrate that the two polyamines control the *V. cholerae* biofilm lifecycle exclusively through NspS-MbaA. Moreover, NspS-MbaA exerts an effect on only a small subset of c-di-GMP-responsive genes, notably *vps* genes, consistent with previous results (17).”

Nevertheless, this should not be perceived as redundancy of the current study with previously published work. On the contrary, the current study does an excellent job of providing explanations to and reconciling many of the observations reported before in the context of nspd/spd signaling through the NspS/MbaA system and the effect of import by PotABCD1 on fine tuning this signaling.

We thank the reviewer for these positive comments.

<u>Minor issues</u>

1. Line 66: Wrong citation. Should be cockerell et al. 2014

   We have corrected this mistake (now line 64).

2. Line 117: Cockerell et al. 2014 shows the norspermidine import by the PotABCD1 transporter and should also be cited.

   We have corrected this mistake.

3. Page 8. The free-energy model assumes that one of the three states MbaA can exist as unbound to NspS and exhibiting DGC activity. Given that the nspS mutant makes very low amounts of c-di-GMP and biofilm, it is unclear to me why MbaA unbound to NspS is thought to have DGC activity. Please provide an explanation that will help the audience understand this assumption.

   We appreciate the reviewer pointing this out. In our model, we allow for the possibility that some MbaA receptors may be in the diguanylate cyclase state while others are in the phosphodiesterase state. The parameter fits to the data determine the abundances of the different states. Using this agnostic fitting strategy allows the model dynamics to be informed more strongly by the experimental reporter data than by upfront assumptions about the allowed protein configurations in different signaling regimes, for which we do not have direct experimental tests. We have clarified this assumption (line 150) to read,

   “In free-energy models, protein configurations, and therefore activities, are drawn from the Boltzmann distribution. Depending on the free-energies associated with each

configuration, which are fit to data, a population of two-state proteins may therefore almost entirely exist in one configuration or the other, or the population may be divided between the two states."

4. Line 255: Please change to "in which nspd production and nspd and spd import" were activated. V. cholerae produces other polyamines (putrescine, diaminopropane, and cadaverine), so the statement as written is inaccurate.

   We clarified the statement as suggested (now line 263).

5. Line 262: Please change to "incapable of nspd production" for the reason stated above.

   Changed as suggested (now line 272)

6. Lines 283-284: Please change to "in which nspd production and nspd and spd import".

   Changed as suggested (now lines 293)

7. Line 287: Please change to "external nspd and spd".

   Changed as suggested (now line 312)

8. Line 297: Similar to above, please specify the type of polyamine import and export

   Changed as suggested (now line 307)

9. Other places in the manuscript where the type of polyamine needs to be specified: lines 300-301, 519 etc.

   As requested, we have clarified cases in which we did not specify which polyamine we meant.

10. Line 519: Please provide information on what is considered "physiologically-relevant" polyamine concentrations.

    We thank the reviewer for bringing up this point. We have clarified the statement to read (line 536),

    "This feature of the NspS-MbaA system presumably allows *V. cholerae* to modify its biofilm lifecycle in response to environmentally encountered spermidine and norspermidine concentrations."

    We do note that, the environmental concentrations of norspermidine are unknown. The point we are making in the manuscript is that a local signaling mechanism allows *V. cholerae* to detect lower norspermidine and spermidine concentrations than it could otherwise, which we presume enables detection and response to these two polyamines even in environmental situations when the two polyamines are relatively dilute.

Reviewer #2:

This study uses a clever combination of computational modelling, biochemistry and genetics to dissect the nspS/mbaA cyclic-di-GMP regulatory circuit in V. cholerae. The authors first construct a free-energy model of the relationship between periplasmic polyamine concentrations and the enzymatic activity of a bifunctional cyclic-di-GMP enzyme; MbaA. Earlier, experimentally derived results for cyclic-di-GMP outputs with different inputs of spermidine and norspermidine alongside biochemical determination of key parameters (Kd etc.) were then used to fit this model. The model successfully recapitulated the behaviour of key V. cholerae mutants, and enabled accurate predictions of states where MbaA / polyamine abundance was perturbed. The authors showed that the NspS-MbaA circuit is exceptionally sensitive to changes in periplasmic levels of norspermidine, and this manifests in downstream cyclic-di-GMP-mediated changes in bacterial behaviour. Polyamine signalling was shown to work exclusively through MbaA, and cyclic-di-GMP produced by MbaA was shown to exclusively control transcription of biofilm genes. The authors go on to test the principles of the global and specific cyclic-di-GMP signalling models, using mbaA alongside other Vibrio DGCs to modulate cyclic-di-GMP and gene transcription. They present evidence that both models probably function in V. cholerae. I have a few comments on the manuscript as it stands:

Major comment

Fig 6: Why does the chromosomal vpvC-W240R gene affect transcription of motility genes, but expressing it in trans to a level that apparently produces the same amount of c-di-GMP does not? On Line 462 the authors state the dynamic range of their system is not enough to achieve the same results seen for the chromosomal mutation, but this seems surprising. DGC genes are not generally expressed at high level, and it seems likely the plasmid borne copy is expressed at higher levels than the chromosomal mutant. Looking at the 1st and 11th columns of the chart in fig 6, the cyclic-di-GMP levels look pretty similar to me. Are these values significantly different from one another?

I suspect that the c-di-GMP measurements seen in the vpvC-W240R mutant and mbaA over-expression strains might be saturating, and do not reflect the true levels of the molecule in these strains. This would explain why vpsL needed to be deleted in these two backgrounds only. If this is the case, then it is difficult to compare the results from these two strains with the results seen for the other strains in this experiment.

This raises a few questions: what is going on with the vpvC-W240R over-expression strain? Did the authors see similar aggregation here? Is this gene really expressed at a lower level in these strains, or is something else, e.g. another, unidentified mutation in the chromosome of vpvC-W240R the cause of these discrepancies?

This could be cleared up by examining the expression levels of the vpvC-W240R gene in these backgrounds by qRT-PCR [or possibly from the RNA seq data]. Likewise, the colony biofilm phenotypes of the expression mutants would be useful to see here or in a supplementary figure. If differential vps-W240R expression cannot explain the different phenotypes seen here, then the authors need to work out what is going on in this strain, possibly by sequencing it and looking for other compensatory mutations that may enhance cyclic-di-GMP levels. Finally, if the c-di-GMP assay is saturating, then the authors should quantify the absolute levels of c-di-GMP in key strains using LC/MS.

We thank the reviewer for bringing up these interesting points. Researchers in the field routinely use *V. cholerae* carrying the *vpvC*^{W240R} mutation as a tool to control c-di-GMP levels. Strains

carrying *vpvC*^{W240R} have elevated c-di-GMP levels compared to the parent *V. cholerae* strain due to increased VpvC activity (see, for example: Jung-Shen B et al. 2021, bioRxiv; Zhang et al. 2021 PNAS; Francisco Díaz-Pascual et al. 2019, Nat Microbiol).

Furthermore, to clarify, our inducible *Pbad-vpvC*^{W240R} construct was not on a plasmid, but rather was integrated in single copy in the chromosome. Also, we note that in the original manuscript, we artificially altered cytoplasmic c-di-GMP levels using chromosomal *Pbad-cdgL* and arabinose induction to ensure that our results were not specific to VpvC^{W240R}.

Regarding reporter saturation in our experiments, the reviewer inspired us to explore whether saturation is an issue in our analyses. To do this, we performed additional experiments. The panel below shows our results. The *V. cholerae* strain carrying the *vpvC*^{W240R} allele at the native *vpvC* locus does not make saturating levels of c-di-GMP. We can elicit higher reporter output by overexpressing *Pbad-vpvC*^{W240R} from a neutral ectopic locus in the strain harboring *vpvC*^{W240R} at the native locus. We have included these data in the revised Supplemental Information as Figure S5.

To make the readers aware that the assay is not saturated, in the main text, we now write.

(line 467) "Importantly, the *V. cholerae* strain carrying the *vpvC*^{W240R} allele does not produce levels of c-di-GMP that saturate the reporter. We know this because we can elicit higher reporter output by additionally overexpressing *Pbad-vpvC*^{W240R} in that strain (S5 Fig)."

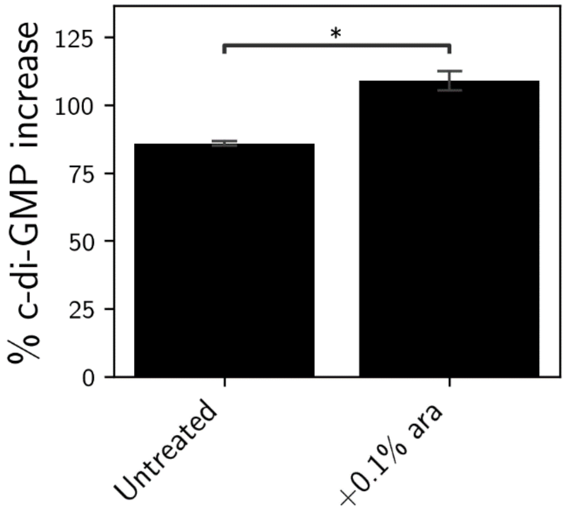

S5 Fig. **The c-di-GMP reporter is not saturated in the *V. cholerae vpvc*^{W240R} strain.** c-di-GMP reporter output from *V. cholerae* carrying *vpvC*^{W240R} at the native *vpvC* locus and *Pbad-vpvC*^{W240R} integrated at an ectopic locus without and with the arabinose inducer as indicated. Data are displayed as percent increases compared to the untreated wildtype strain. A Kruskal-Wallis test was performed for statistical analysis. *0.01 < $P \leq 0.05$; n.s., $P > 0.05$.

Finally, the reviewer makes the point that one might expect VpvC^{W240R} levels to be higher in the strain carrying inducible *Pbad-vpvC*^{W240R} under conditions of high inducer concentrations compared to the strain carrying the allele expressed from the endogenous locus. Indeed, the reviewer is correct. RNA sequencing shows that chromosomal *vpvC*^{W240R} expression is slightly lower than that from *Pbad-vpvc*^{W240R}. The observed differences in downstream gene expression (motility/biofilm) between the endogenous *vpvC*^{W240R} background and the inducible *vpvC*^{W240R} are due to induction time. The endogenous VpvC^{W240R} strain constitutively produces c-di-GMP and

accumulates it over many generations, whereas the inducible strain only begins to increase c-di-GMP when we add inductant (at the start of the experiment), shortly before collecting cells for measurements. To clarify this issue, we have included the following statement in the text:

(line 480) "Due to short induction times, the arabinose inducible *vpvC*^{W240R} construct did not enable us to achieve the sustained high c-di-GMP concentrations needed for repression of motility genes."

Minor points:

1. Line 41: Ensure the VPS abbreviation is explicitly defined here.

We have changed the text to read:

(line 38) "… the VpsT and VpsR transcription factors bind to c-di-GMP and subsequently activate expression of vibrio polysaccharide biosynthesis (*vps*) genes (11). Biosynthesis of the Vps matrix promotes biofilm formation."

2. Line 82 onwards: I don't think this can be stated as an either/or question. There is strong evidence for both delocalized effector affinity [https://onlinelibrary.wiley.com/doi/full/10.1111/mmi.12066] and localized signalling networks [https://journals.asm.org/doi/10.1128/mBio.01639-17] operating in different bacterial contexts. The authors should consider rephrasing this section more towards an assessment of the relative importance and potential overlap of these different mechanisms, rather than presenting a binary choice between them.

We have changed this passage accordingly to state:

(line 84) "We address the long-standing issue of whether c-di-GMP signaling specificity is a consequence of localized c-di-GMP transmission between specific pairs of receptors and effectors, and/or, if changes in the global cytoplasmic c-di-GMP pool are detected by particular effectors based strictly on their relative affinities for c-di-GMP (14)."

4. Figure 5E: The legend description for this panel is rather ambiguous and should be clarified. What does this plot represent? I guess this is mutant vs WT?

We thank the reviewer for noticing this oversight, the revised legend reads as follows:

(line 401) "(E) As in A, for the *V. cholerae vpvC*^{W240R} strain, fold changes are relative to the untreated WT strain."

5. Line 389-408 and Fig 5E: Point mutants in other DGC enzymes (e.g. WspR19 in Pseudomonas, PleD* in Caulobacter) have been shown to induce overproduction of c-di-GMP far in excess of the physiological maximum for the system in nature, typically by disabling product inhibition. The authors need to show evidence that this is, if not impossible here, then at least unlikely. The authors should state what is known about the activation mechanism of the W240R mutation, from the earlier work of Beyhan and Yildiz.

To our knowledge, the maximum concentration of c-di-GMP in *V. cholerae* in nature is not known. As noted above, we are using the *vpvC*^{W240R} mutation simply as a tool to allow us to control/modulate c-di-GMP levels, as have many *V. cholerae* researchers before us. In the present work, we are not investigating the biological role of VpvC. Likely it is fascinating, but that role does not have direct bearing on our present studies.

5. Line 409-428: The extensive description of these two signalling models would be better placed in the discussion. The models could be briefly introduced here for the purposes of continuity with the following section, but then discussed more thoroughly later.

With respect, differentiating between local and global signaling is one of the main questions under study in this work. Thus, our view is that it is critical to establish the issue being probed, the logic motivating the experimental approaches, and the results we garner that speak to the question, prior to bringing up the topic in the Discussion. Doing so is especially important for less expert readers who might not know the outstanding questions.

6. Fig 6: The scale on the bottom panel doesn't make sense to me. If these values are relative to WT, then how can they be expressed as both a positive and a negative percentage of reporter output?

To clarify, the reporter outputs are all normalized to the WT output as percentage differences. For example, addition of norspermidine increases c-di-GMP output relative to the untreated strain, while treatment with spermidine decreases reporter output relative to the untreated WT. To ensure that readers are not confused, we clarified the legend as follows:

(line 499) "Bottom panel: Mean global c-di-GMP reporter outputs for the indicated strains and conditions expressed as percentage differences relative to untreated wildtype *V. cholerae*."

Reviewer #3:

This is a very interesting manuscript addressing a pending question about how the information lying into the universal second messenger c-di-GMP is able to be transduced into multiple and specific responses in bacteria. Here the authors present a combination of experimental and modelling data, using one of many diguanylate cyclases of Vibrio cholerae, MbaA, and testing how the response to polyamine, via NsbS-MbaA interaction, is effectively transduced, locally or globally and with a general or specific impact on Vibrio physiology, notably biofilm formation.

Although I have no appropriate expertise to assess the buildup of the mathematical modeling, the biology presented holds a number of novel concepts, or provides previously accepted concepts with experimental validation here. In general, the paper is rather dense and somehow complex, although the concepts that are conveyed are straightforward.

Here below are a few comments that mostly relates to the biological aspect of the work:

- The authors showed that in absence of production (nspC mutant) and import of polyamine (potD mutant) the detection of exogenously added norspermidine is highly sensitive (sub-nanomolar range) as monitored by biofilm formation (line 308). Biofilm is also driven through production of the VPS polysaccharide. Subsequently the authors test the reporter vpsL-lux against the addition of polyamine to wild-type Vibrio and observed that addition of micromolar range of norspermidine has no effect on global c-di-GMP but does impact biofilm and vpsL-lux expression. This led the authors to conclude to a local mechanism of transmission of c-di-GMP signaling, whose variation in concentration is not seen at the global level, but still effects a specific response on VPS. i) It would be appropriate to test whether in a npsC/potD mutant, and not a wild-type, the variation in level of c-di-GMP is observed, and that cannot be seen in the wild-type due to depletion of the periplasmic norspermidine upon its transport into the cytoplasm.

We thank the reviewer for this suggestion. We agree that it is crucial to test whether, in the Δ*nspC* Δ*potD1* strain, low levels of supplied norspermidine elicit changes in global c-di-GMP levels that do not occur in the wildtype strain. We note that these data were provided in the original manuscript (Fig. 3B-D). Indeed, as the reviewer suspects, sub-nanomolar concentrations of norspermidine drive an increase in global c-di-GMP levels in the Δ*nspC* Δ*potD1* strain, whereas ~10-50 μM norspermidine is required for such a response in the wildtype.

ii) It would also be appropriate to test whether the response is c-di-GMP-dependent in the wild type by using a mutant in which the GGDEF motif of MbaA has been mutated so that there is no longer diguanylate cyclase activity. This way it confirms that the despite the lack of global change in c-di-GMP it is a c-di-GMP-dependent response that is observed.

We thank the reviewer for this suggestion for an excellent experiment, which we performed. Below, and in the revised Supplemental Information (S3 Fig), we provide a new figure with the requested data. Using an MbaA mutant that is defective for c-di-GMP biosynthesis, we demonstrate that polyamine-driven *vpsL-lux* output depends on MbaA diguanylate cyclase enzymatic activity.

S3 Figure

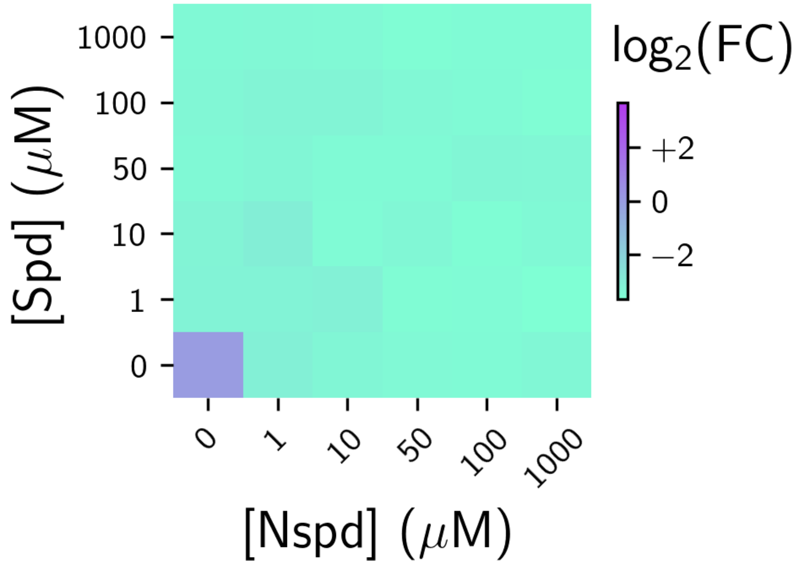

S3 Figure. **Induction of *vps* expression by polyamines requires MbaA diguanylate cyclase activity.** Shown is the *vpsL-lux* reporter output for MbaA carrying the D426A and E427A substitutions. In this mutant MbaA protein, the SGDEF catalytic site is altered to SGAAF which eliminates c-di-GMP biosynthetic capability. Data are displayed as $\log_2$ fold-changes relative to the untreated strain (bottom left corner).

We refer readers to the new figure and data with the following text:

(line 336) "These changes depended on MbaA possessing c-di-GMP biosynthesis capability, as norspermidine and spermidine did not elicit alterations in *vpsL* expression in an MbaA SGAAF mutant that is defective for c-di-GMP biosynthesis (S3 Fig)."

- One assumption that comes out of the work (line 544) is that a small fraction of NsbD unbound to norspermidine (apo-NspS) would interact with MbaA. Could the authors discuss whether this reflects a difference in affinity between the close and open state of NspS. Would it be possible that NspS bound to spermidine also binds at low frequency to MbaA and how will this be accounted for into the mathematical model?

We thank the reviewer for this point. The small amount of NspS-MbaA binding in the absence of norspermidine in the model does not reflect a difference in the affinities of the closed- and open-states of NspS for MbaA, but rather, the fraction of NspS in the closed-state when norspermidine is absent. Specifically, the results of our parameter fits suggest that, in the absence of polyamines, a small fraction of NspS exists in the closed state, and this small fraction is able to bind to a fraction of the MbaA molecules and induce their diguanylate cyclase activity. To be clear, we assume that only NspS in the closed state is capable of binding MbaA. Therefore, open-state NspS has no affinity for MbaA, so NspS bound to spermidine, which is in the open state, cannot bind to MbaA. We note that this information is in the manuscript. Additionally, below and in the revised manuscript, we provide a new figure and associated text showing that if NspS and MbaA are overexpressed from their native loci using the *Ptac* promoter, the level of c-di-GMP reporter

output increases dramatically, even in the absence of norspermidine (Fig. S1F). These data suggest that apo-NspS can bind MbaA and drive diguanylate cyclase activity.

(line 193) "To further test this prediction, we overexpressed *nspS* and *mbaA* from the native locus using the *Ptac* promoter, and we measured c-di-GMP reporter output across varying levels of norspermidine and spermidine. c-di-GMP reporter output was strikingly elevated compared to that in the wildtype strain, including in the absence of norspermidine. This result suggests that apo-NspS can bind MbaA and elicit diguanylate cyclase activity."

S1F Figure

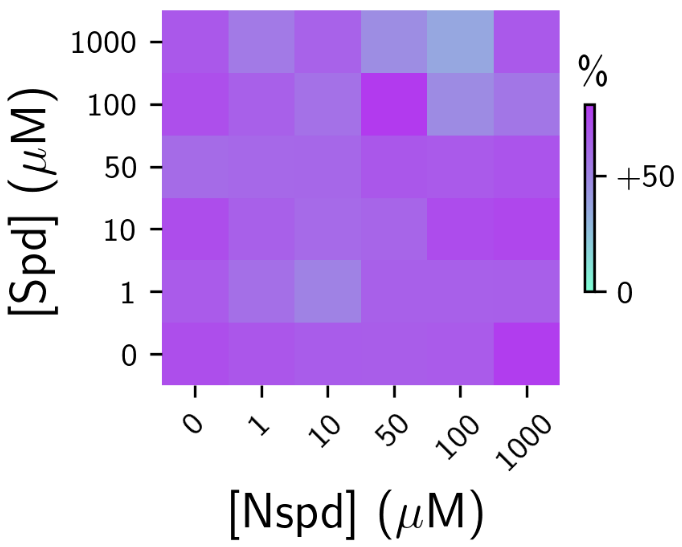

S1 Fig. (F) Shown is the c-di-GMP reporter output for a *V. cholerae* strain carrying *Ptac-nspS-mbaA* at the native *nspS-mbaA* locus. Data are displayed as percent increases relative to the wildtype strain with no polyamines added.

- Lines 159-160 it is indicated that MbaA unbound to NspS can also display diguanylate cyclase activity. I am not sure to clearly grasp what is the rationale behind this. Could the authors clarify? Does purified MbaA have cyclase or phosphodiesterase activity? Which activity, cyclase or phosphodiesterase, does a truncated MbaA carrying only the GGDEF and EAL domain have?

We thank the reviewer for this point. See also point 3 of Reviewer #1.

In our model, we allow for the possibility that some MbaA receptors may be in the diguanylate cyclase state while others are in the phosphodiesterase state. The parameter fits to the data determine the abundances of the different states. Using this agnostic fitting strategy allows the model dynamics to be informed more strongly by the experimental reporter data than by upfront assumptions about the allowed protein configurations in different signaling regimes, for which we do not have direct experimental tests.

We have clarified this assumption (line 150) to read,

"In free-energy models, protein configurations, and therefore activities, are drawn from the Boltzmann distribution. Depending on the free-energies associated with each configuration, which

are fit to data, a population of two-state proteins may therefore almost entirely exist in one configuration or the other, or the population may be divided between the two states."

- It would be appropriate to briefly comment on how the NspS-MbaA system can be adapted to other c-di-GMP signaling systems as mentioned on line 565.

We thank the reviewer for this suggestion. In the revised manuscript, we have now included text concerning the relevance of the NspS-MbaA circuit to other c-di-GMP signaling systems (lines 582):

"Nevertheless, for c-di-GMP metabolizing receptors with known ligands, such as VC1086 and CdpA (nitric oxide), VC1710 (sugars), and CdgH (arginine) in *V. cholerae*, combining mathematical modeling with measurements of changes in c-di-GMP levels and downstream transcriptional reporter assays could be immediately undertaken and possibly reveal the interactions driving the observed input-output relations (28–31)."

Reviewer #4:

In this submission, Bridges, Bassler and colleagues continue their investigation in the polyamine-sensing system controlling intracellular c-di-GMP in Vibrio cholerae, whose key components are the NspS polyamine periplasmic receptor and its binding partner, the inner-membrane bifunctional diguanylate cyclase/phosphodiesterase enzyme MbaA. In contrast to other inner-membrane, ligand-sensing c-di-GMP regulatory systems, the ligands for the NspS/MbaA partners have been identified and depending on the specific bound polyamine and related ligand- and protein partner-binding affinities, the system can switch from c-di-GMP generation to c-di-GMP degradation with the associated inverse effects on biofilm formation and dispersal, respectively.

The manuscript is clearly written and easy to follow and the data representation, as typical for this group's works, is neat and self-explanatory. The experiments are well controlled and logical and overall of good quality. The authors present simple and intuitive mathematical models that however satisfactorily describe the observed effects of ligand modulation on c-di-GMP levels and biofilm formation. If anything, the text can profit from a better introduction of the role of spermidine and norspermidine on the pathogen's physiology and in particular in relation with biofilm formation vs. dispersal in the environment and the host. The physiological concentration ranges for the two polyamines, if known, would be also very relevant to refer to throughout the study.

My major concern about this article is that it is quite incremental with regard to recent findings by the same group published elsewhere and in particular the Bridges & Bassler eLife paper from a few months ago where the system's workings were reported with regard to biofilm dispersal, a process intrinsically inverse to biofilm formation. The mechanism and effects of norspermidine vs spermidine sensing via NspS, MbaA and even associated partners involved in polyamine import were already beautifully reported in that study. From the underlying hypotheses, to the specific experimental toolkit and examined mutants, the current manuscript is mostly an intuitive continuation of the previous study. While the current submission provides a more quantitative rationalization of the observed effects, overall I don't find it provides substantial new insights into the mechanisms of Vibrio biofilm formation.

What seemed potentially interesting is the possibility to distinguish between local signal transduction through direct generation-sensing-degradation of c-di-GMP among interacting or spatially constrained proteins vs. modulation of the global pool of available c-di-GMP. Overall, however, I feel that the manuscript falls short in doing that.

If I am not wrong many, if not most, c-di-GMP signaling systems experience some degree of pathway specificity which is in line with the presence of multiple non-redundant DGCs, PDEs or bifunctional enzymes per genome, including that of V. cholerae. For example, work from the Hengge lab and others have shown that secretion of biofilm matrix components such as polysaccharides and curli in enterobacteria (e.g. E. coli and Salmonella) is often controlled by a multilevel cascade of c-di-GMP sensing proteins and interacting DGCs, PDEs and even proteins directly involved in secretion. Yet, almost any active DGC induced in a standard E. coli protein expression strain that barely secretes extracellular polymers can lead to secretion of biofilm matrix components circumventing both pathway-specific enzymes and spatial restrictions (which is actually routinely used as an assay for identification of enzymatically active DGCs). The point being that the biofilm-stimulating effects observed by non-specific modification of global c-di-GMP levels in Vibrio here, even upon adjusting for cellular c-di-GMP concentrations with regard to the NspS/MbaA system are not particularly surprising, especially since the read-out of cytosolic c-di-GMP concentrations is itself determined by c-di-GMP complexation by the used reporter.

Interestingly, the authors do observe some specific effects at low and likely more physiological polyamine concentrations where the global c-di-GMP concentration does not exhibit significant changes (again, dependent on the used reporter).

The key question, and answer, here that can bring substantial novelty to the work and make it suitable for publication in PLoS Biology would be what are the local/pathway-specific effectors that could contribute to physiological polyamine sensing and related MbaA activities? The authors offer several specific leads in the discussion yet none of these were experimentally tested and overall the results and discussion related to the local vs global c-di-GMP sensing remain mostly handwaving. For example, VpsT is known to complex c-di-GMP and form supramolecular clusters that can be somewhat easily observed in spermidine vs norspermidine exposure and may be co-localized with the proteins in question, even if high-affinity interactions do not necessarily occur stably. Protocols for expression (and purification) of both VpsT and VpsR, two biofilm transcriptional regulators and potential MsbA partners discussed by the authors are available and could be tested for interactions with the system's components both in cell-based and in vitro assays that seem well within the expertise of the group. Of course, an unbiased protein partner screening approach would be preferred as biofilm-promoting effectors are not necessarily part of the known transcription regulators.

Somewhat minor comments: it seems the effects on biofilm-stimulating genes is more pronounced than those on flagellar motility genes even in the context of very high cytosolic c-di-GMP, so maybe it is not surprising that the biofilm genes are the first to be detected upon subtle changes in the polyamine concentrations/MbaA activity changes. As the authors only looked at transcriptional changes, whereas local signaling effects can be also exerted on already expressed effectors (i.e. post-translationally), differential effects would be also dependent on the target promoters and not only on the c-di-GMP-binding affinities of the involved transcriptional regulators. These caveats should be considered in the models and discussion.

In conclusion, this article provides additional examination of a system already quite well characterized by the Bassler group at the level of spermidine/norspermidine sensing and the inverse effects of the two polyamines on MbaA activity and downstream biofilm effects. It pains me to write this, but I do not believe the novelty and insights gained here are nearly sufficient for a publication in PLoS Biology or another novelty-driven PLoS journal unless further data on the downstream signal effectors are indeed reported.

We thank the reviewer for his/her comments, but we respectfully disagree that the current work overlaps with, or does not provide substantial new insights, relative to our previous study. Indeed, the components and steps of the pathway were identified already, as the reviewer states. Such is the case for many bacterial and eukaryotic signaling systems. Identifying components is typically the first, not the final step that launches new research areas. Additional investigations are warranted to understand function. For example, the components of the *E. coli* chemotaxis pathway have been known for nearly 60 years, yet thousands of fascinating papers have been and continue to be published in journals with an emphasis on novelty since initial component identification, and our understanding of chemotactic behavior has achieved great depth because of that.

With respect to the NspS-MbaA pathway prior to the present work, there was no quantitative investigation of c-di-GMP signal transmission to explain system input-output dynamics. There was no molecular explanation for the observed and unexpected polyamine responses. There was no molecular explanation for the effects of PotABCD1-mediated import on signal transduction. There

was no modeling. There was no measurement of the influence of signaling on c-di-GMP levels. All of those facets, and others, are provided for the first time in this manuscript.

We wholly agree with the reviewer is that a next goal is to identify the effector through which NspS-MbaA signals. However, that is beyond the scope of the current manuscript as, and the reviewer notes this, doing that demands identification of interaction partners, purification and testing of interactions in vitro, localization studies and likely mutagenesis.

Regarding specific changes requested by the reviewer:

We have improved our introduction of the roles spermidine and norspermidine play in physiology, and in particular in biofilm formation and dispersal. We have included the following statement and citations:

(line 68) "Because norspermidine is a rare polyamine in the biosphere whereas spermidine is widely produced across domains (and is present in the mammalian intestine at micromolar concentrations), the hypothesis is that the two polyamines allow *V. cholerae* to decipher the relatedness of other bacterial species in the vicinal community (16,18,20–22). When closely related species are detected (via norspermidine) the biofilm lifestyle is favored, whereas when non-related species are detected (via spermidine), dispersal occurs and *V. cholerae* commits to the planktonic state, presumably to flee competitors."

The reviewer makes the excellent point that, in a global signaling mechanism, specificity could be achieved through affinity differences between downstream effectors and their target promoters. In the revised manuscript, we now include this possibility in the following statement in our text about global and local c-di-GMP signaling:

(line 426) "First is the "global signaling model" (Fig 5F, left panel), in which the c-di-GMP produced by a given diguanylate cyclase freely diffuses throughout the cytoplasm. Specificity in target gene expression is achieved by differences in affinities of downstream effectors for c-di-GMP, and/or differences in effector affinities for target promoters (14)."
